# Shape Matters: Long-Range Transport of Microplastic Fibers in the Atmosphere

**DOI:** 10.1021/acs.est.3c08209

**Published:** 2023-12-27

**Authors:** Daria Tatsii, Silvia Bucci, Taraprasad Bhowmick, Johannes Guettler, Lucie Bakels, Gholamhossein Bagheri, Andreas Stohl

**Affiliations:** † Department of Meteorology and Geophysics, University of Vienna, Universitätsring 1, 1010 Vienna, Austria; ‡ Laboratory for Fluid Physics, Pattern Formation and Biocomplexity, Max Planck Institute for Dynamics and Self-Organisation, Am Faßberg 17, 37077 Göttingen, Germany; § Institute for the Dynamics of Complex Systems, University of Göttingen, Friedrich-Hund-Platz 1, 37077 Göttingen, Germany

**Keywords:** microplastics, shape, atmospheric transport, gravitational settling, fibers

## Abstract

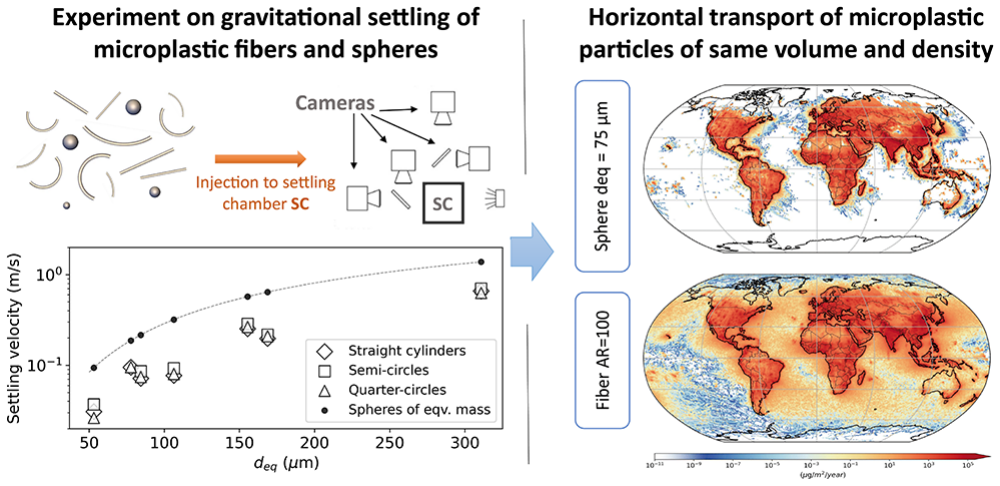

The deposition of airborne microplastic particles, including those exceeding 1000 μm in the longest dimension, has been observed in the most remote places on earth. However, their deposition patterns are difficult to reproduce using current atmospheric transport models. These models usually treat particles as perfect spheres, whereas the real shapes of microplastic particles are often far from spherical. Such particles experience lower settling velocities compared to volume equivalent spheres, leading to longer atmospheric transport. Here, we present novel laboratory experiments on the gravitational settling of microplastic fibers in air and find that their settling velocities are reduced by up to 76% compared to those of the spheres of the same volume. An atmospheric transport model constrained with the experimental data shows that shape-corrected settling velocities significantly increase the horizontal and vertical transport of particles. Our model results show that microplastic fibers of about 1 mm length emitted in populated areas are more likely to reach extremely remote regions of the globe, including the high Arctic, which is not the case for spheres of equivalent volume. We also calculate that fibers with lengths of up to 100 μm settle slowly enough to be lifted high into the stratosphere, where degradation by ultraviolet radiation may release chlorine and bromine, thus potentially damaging the stratospheric ozone layer. These findings suggest that the growing environmental burden and still increasing emissions of plastic pose multiple threats to life on earth.

## Abstract 2

This study shows that microplastic fibers have a large atmospheric transport potential, which is explained by a newly proposed method for atmospheric transport models.

## 1 Introduction

Microplastics (MP) are present ubiquitously, and their harmful effects on the environment are substantial. Microplastics are synthetic organic polymers of various shapes and colors with the size of the largest dimension ranging from 1 μm to 5 mm.^1^ They can be intentionally produced (e.g., as abrasives) or are a product of breaking of larger plastic particles via weathering or UV-radiation exposure.^2^ The presence of MP is reported in diverse environmental compartments;^3^ however, the knowledge about their abundance in the atmosphere is still limited.^4^ As indicated by Dris et al.^5^ and Cai et al.,^6^ airborne microplastics have the potential to contaminate both terrestrial and aquatic ecosystems. Furthermore, there is a possibility of direct inhalation and subsequent accumulation of microplastics in the lungs of humans.^7^ Therefore, it is crucial to know their distribution in the global atmosphere, for which an accurate understanding of their emissions and removal mechanisms is essential.

Accumulation mode particles of different species sized 100–1000 nm are known to be transported over long distances in the atmosphere, sometimes across an entire hemisphere.^8^ Several studies^9−14^ have found that even particles larger than 75 μm in diameter can stay airborne for extended periods and be deposited thousands of kilometers away from their sources. This also applies to microplastics, which are often found in remote regions of the world.^15−20^ However, the long-range transport is difficult to reproduce with current atmospheric transport models, which predict a much shorter dispersion range for such large particles.^21,22^ The question is what mechanisms or model deficiencies can explain these discrepancies.

Several mechanisms that have been suggested to enhance transport distances, for instance, strong winds,^23^ strong turbulence keeping individual particles aloft,^24^ or electric forces counteracting the particles’ weight^25,26^ seem insufficient to resolve the model problems. Another possibility investigated in this paper is the effect of the particle shape. Most regional or global transport models treat particles as perfect spheres, whereas in reality, their shapes are often far from spherical. Such particles experience a larger drag in the atmosphere compared to spheres, which reduces their settling velocity and facilitates longer transport distances.^27^ This is particularly true for microplastics, which are often found as fibers or with other complex shapes in the environment.^15−20^

To date, shape corrections for gravitational settling calculations^28^ have been mostly used for modeling the dispersion of volcanic ash^27^ and mineral dust.^29^ However, microplastic fibers with lengths that exceed their thickness by a factor of 40 or more represent a greater challenge. While their settling behavior in water or other liquids is already relatively well-constrained, only a few experiments have investigated the settling of nonspherical particles in air,^28,30−32^ and the available data do not cover the range of sizes and shapes relevant to microplastics. In particular, data on the settling behavior of fibers, especially of bent shapes, are missing, and this limits our capability to reliably simulate the dispersion of microplastics in the atmosphere.

Therefore, the aim of this study is to determine the settling velocities of microplastic fibers of different sizes and shapes by performing a series of experiments in a newly developed laboratory setup.^30^ In addition, we investigate the influence of particle orientation on the settling velocity, which can be an important parameter in the transport of particles in the turbulent atmosphere. The model is then used to constrain a global atmospheric transport model and analyze the impact of gravitational settling on the global microplastic transport in the atmosphere.

## 2 Methods

### 2.1 Experiments on the Settling Behavior of Fibers

We investigated the free-fall behavior of MP, specifically MP fibers in air, using a novel, high-precision experimental setup, the Göttingen turret (see “Experimental Setup”). To date, only a small number of experiments on the gravitational settling of MP fibers have been reported.^31−33^ The experiment in this study distinguishes itself by its unique setup, advanced technologies in terms of optics, and precisely defined fibers with a much wider range of shapes and sizes.

#### 2.1.1 Fiber Manufacturing and Characterization

The fibers were produced using two-photon (2P) lithography technology, used for three-dimensional (3D) polymer structure printing. The Photonic Professional (GT) by the Nanoscribe 3D printer^34^ was utilized for this purpose. We used the acrylic resin IP-S photoresist (CH_1.72_N_0.086_O_0.37_),^35^ which was polymerized by the 3D printer using a 25× objective with a 780 nm laser to an intended shape. In this setup, surface features of the fibers were printed with sub-μm accuracy,^34^ and after polymerization, the printed fibers had a density of ρ_p_ = 1200 kg/m^3^.^35^ The density of the printed fibers is similar to the densities of most common microplastic polymers [poly(ethylene terephthalate) (PET), high-density polyethylene (HDPE), polypropylene (PP), polystyrene (PS), etc.]. To uniformly reproduce the curvature in the shape of the cylindrical fibers, we printed the fibers in multiple solid layers stacked on top of each other. The thickness of such solid layers (slicing distance) is 0.4 μm, while each layer is printed as a combination of voxels with a volume of 0.3 × 0.3 × 0.4 μm^3^ (more details in refs (30) and (34)).

**Figure 1:**
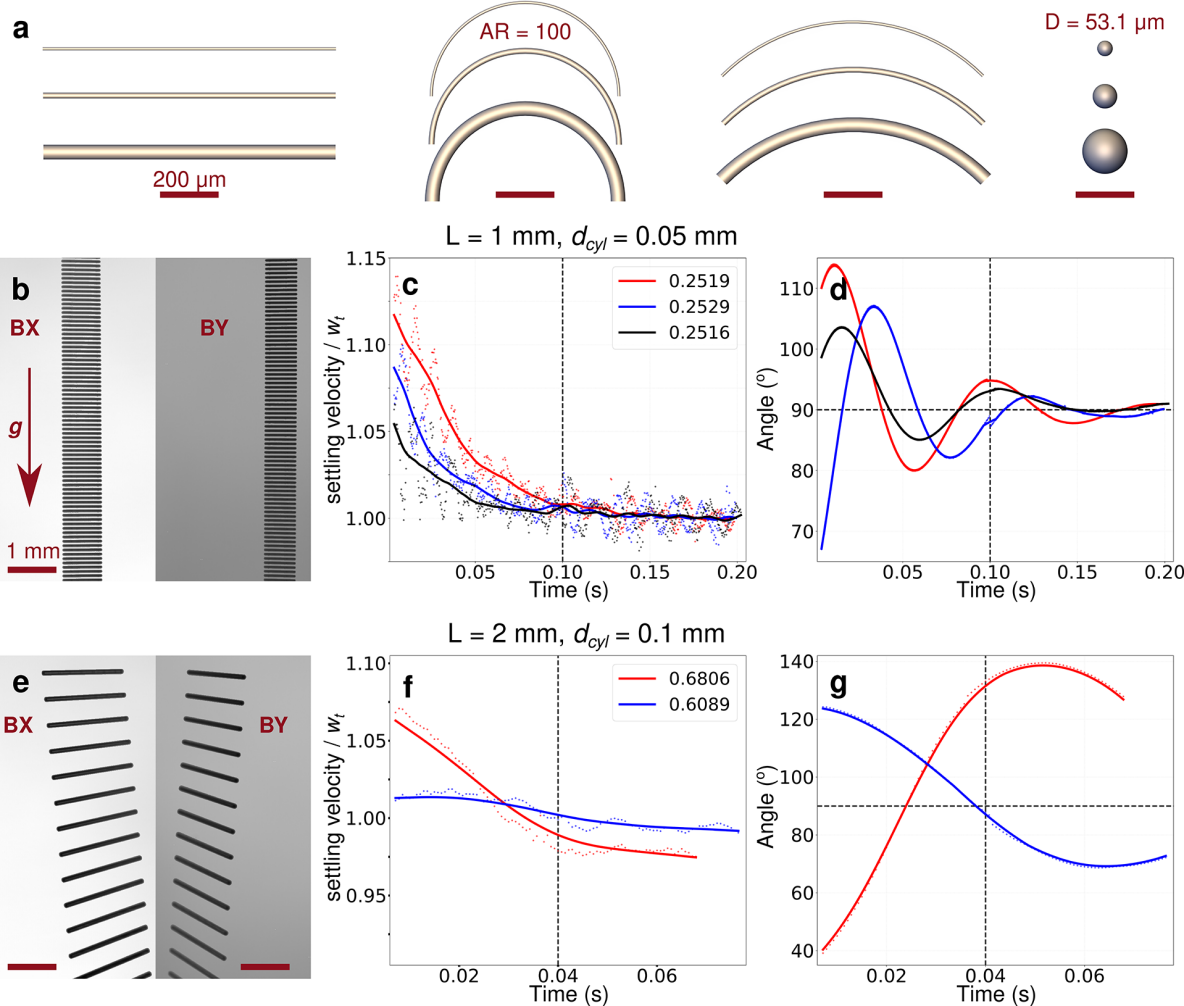
Example of the particles printed for the experiments and the settling behavior of fibers. (a) Straight, semicircular, and quarter circular 1 mm fibers for different aspect ratios AR and the corresponding diameters *d*_eq_ of volume equivalent spheres (sizes of other printed particles are listed in Table 1). Experimental recording of the gravitational settling behavior of straight fibers of (b) 1 and (e) 2 mm length (*L*) with an aspect ratio (AR) of 20. The entire recording of the upper cameras TX and TY and the bottom cameras BX and BY is shown in Figure S4, and *g* shows the gravity direction. The postprocessed time series of (c,f) settling velocity and (d,g) orientation, respectively, for 1 and 2 mm straight fibers from the entire recording. The dots represent the experimental postprocessed data for each camera image, and the averaged experimental data are shown as solid lines. The left half of the vertical dashed lines contains the data from the upper cameras (TX and TY), while the right half corresponds to the same from the bottom cameras (BX and BY). The settling velocity in (c,f) is normalized to the terminal velocity *w*_t_ (average of the settling velocity from the bottom cameras), which is given in units of m s^–1^ in the legend. The horizontal dashed line in (d,g) corresponds to the steady-state orientation of the fiber, i.e., 90°.

According to existing images of deposited MP particles, MP fibers can be straight, curved threads, or spirals.^36−40^ Hence, we decided to print out MP fibers of cylindrical shape with different degrees of curvature: straight, semicircular, and quarter circular. For the drag model, the dimensions of the fibers are defined by the longest axis *L* (the length of the straight fiber or the distance between the cylinder’s edges for the curved cylinders) and the smallest axis *S* (the diameter of these cylindrical fibers, d_cyl_), following the minimum bounding-box principle.^41^*L*, *I*, and *S* are shown in Figure S1. We printed MP fibers with lengths of 500, 1000, and 2000 μm, each with aspect ratios AR (i.e., length/*d*_cyl_) of 20, 50, and 100. The sphericity Ψ, which is a measure of the surface area of a particle compared to that of a sphere of the same volume with values ranging from 0.0 (less spherical) to 1.0 (spherical),^41^ varies from 0.28 to 0.47 for printed fibers (Table 1). Sphericity is defined as Ψ = π*d*_eq_^2^/SA, where *d*_eq_ is the volume equivalent diameter of a sphere and SA is the surface area of a particle. Renderings of these fibers are shown in Figure 1a and microscopic images of actual printed fibers were obtained with a Keyence VK-X200 K laser microscope (Figure S1).

**Table 1:** Characteristics of the Printed Particles and Their Measured and Estimated Settling Velocities *w*_t_^a^

shape	name	*d*_eq_ (μm)	length (μm)	*d*_cyl_ (μm)	Ψ	*f*	*e*	*N*	*w*_t,exp_ (m/s)	σ_*w*t,exp_ (m/s)	*Re*	*w*_t,aver_ (m/s)	*w*_t,max_ (m/s)	*w*_t,rand_ (m/s)	*w*_t,sph_ (m/s)
straight fiber	c1	310.7	2000	100.0	0.46	1.000	0.050	11	0.6625	0.0132	13.35	0.6573	0.5763	0.7770	1.3860
	c2	168.7	2000	40.0	0.35	1.000	0.020	6	0.1983	0.0002	2.44	0.2212	0.1967	0.2560	0.6448
	c3	106.2	2000	20.0	0.28	1.000	0.010	6	0.0775	0.0010	0.57	0.0816	0.0728	0.0927	0.3201
	c4	155.3	1000	50.0	0.47	1.000	0.050	11	0.2535	0.0003	2.70	0.2654	0.2347	0.3068	0.5734
	c5	84.3	1000	20.0	0.35	1.000	0.020	5	0.0718	0.0002	0.39	0.0707	0.0631	0.0800	0.2168
	c6	53.1	1000	10.0	0.28	1.000	0.010	4	0.0309	0.0012	0.08	0.0227	0.0204	0.0257	0.0940
	c7	77.7	500	25.0	0.47	1.000	0.050	4	0.0953	0.0012	0.44	0.0856	0.0767	0.0974	0.1881
															
semicircular fiber	sc1	310.7	2000	100.0	0.46	0.157	0.500	7	0.7043	0.0148	12.60	0.6203	0.5510	0.7360	1.3860
	sc2	168.7	2000	40.0	0.35	0.063	0.500	12	0.2179	0.0010	2.54	0.2302	0.2031	0.2555	0.6448
	sc3	106.2	2000	20.0	0.28	0.031	0.500	9	0.0928	0.0006	0.69	0.0996	0.0898	0.1088	0.3201
	sc4	155.3	1000	50.0	0.47	0.157	0.500	10	0.2882	0.0093	2.85	0.2810	0.2505	0.3272	0.5734
	sc5	84.3	1000	20.0	0.35	0.063	0.500	3	0.0859	0.0029	0.49	0.0889	0.0787	0.0984	0.2168
	sc6	53.1	1000	10.0	0.28	0.031	0.500	2	0.0371	0.0027	0.12	0.0335	0.0301	0.0369	0.0940
															
quarter circular fiber	qc1	310.7	2000	100.0	0.46	0.284	0.197	9	0.6309	0.0055	12.27	0.6042	0.5323	0.7137	1.3860
	qc2	168.7	2000	40.0	0.35	0.108	0.208	4	0.2094	0.0004	2.37	0.2153	0.1940	0.2463	0.6448
	qc3	106.2	2000	20.0	0.28	0.053	0.211	6	0.0801	0.0002	0.63	0.0904	0.0803	0.0981	0.3201
	qc4	155.3	1000	50.0	0.47	0.284	0.197	12	0.2652	0.0004	2.68	0.2635	0.2320	0.3036	0.5734
	qc5	84.3	1000	20.0	0.35	0.108	0.208	6	0.0744	0.0003	0.44	0.0794	0.0718	0.0903	0.2168
	qc6	53.1	1000	10.0	0.28	0.053	0.211	2	0.0263	0.0	0.10	0.0262	0.0257	0.0318	0.0940
	qc7	77.7	500	25.0	0.47	0.284	0.197	3	0.0955	0.0003	0.47	0.0924	0.0825	0.1049	0.1881
															
sphere	s1	185.0			1.00			6	0.6980	0.0146	8.84				0.7314
	s2	107.0			1.00			4	0.3267	0.0025	2.26				0.3237

a Here, *d*_eq_ is the volume equivalent diameter of a sphere, *d*_cyl_ is the diameter of a fiber, length is the actual length of the fiber, Ψ is sphericity, *f* is flatness, and *e* is elongation.^41^*N* is the number of successful experiments. Values for *w*_t,exp_ are given as averages over the number of experiments of corresponding shape and size, with σ_*w*t,exp_ being the standard error. *w*_t,aver_, *w*_t,max_, *w*_t,rand_, and *w*_t,sph_ are modeled settling velocities for the averaged, maximum-drag, random orientations of falling, and for volume equivalent spheres, respectively.

For each fiber type, we printed 25–60 identical fibers to facilitate repeated experiments. The fiber sizes and shapes are listed in Table 1. We checked all printed fibers for irregularities with the laser microscope and removed the ones that had deformations. More complex fibers such as spirals or fibers with smaller *L* values were not considered due to the limitations of the 3D printer, the high fragility of such fibers, the difficulties in successfully separating and injecting them into the settling chamber, and the size limitations imposed by the resolution of the cameras used to record the settling behavior of the fibers.

#### 2.1.2 Experimental Setup

The experimental setup (the Göttingen turret), which is used to measure the settling velocities of the fibers, is described in detail in Bhowmick et al.^30^ (shown in Figure S2). This setup is composed of a novel particle injector, an air-filled settling chamber, and four high-speed cameras synchronized together with a high-intensity LED array, in which one can currently measure the transient settling dynamics of solid particles in the size range of 0.1–5 mm in quiescent air. This setup also consists of a Photonic Professional GT two-photon polymerization 3D printer with nanometer-micrometer resolution capable of printing highly reproducible particles of a specified shape as per requirement. The air-filled settling chamber has a dimension of 90 × 90 × 200 mm in the *X*, *Y*, and *Z* directions, into which the fibers are inserted with the particle injector and allowed to settle under gravity. The settling chamber is imaged by four high-speed cameras (Phantom VEO4K 990L, Vision Research) with each image pixel corresponding to a physical area of 6.75 × 6.75 μm^2^ in a camera field-of-view of 27 650 μm vertical and 3510 μm horizontal extent (i.e., 4096 × 520 pixels) and is illuminated by an LED lamp (LED-Flashlight 300, LaVision GmbH).

The aim of the experiments was to observe the settling behavior of the fibers in their steady state. Therefore, the settling chamber had to be brought to such a vertical height that the cameras could see the fibers in their steady state. The model of Bagheri and Bonadonna (2016)^28^ is used to determine the vertical distance at which the fibers reach approximately their steady-state settling velocity. After the vertical position of the settling chamber was set, the setup was calibrated prior to the experiments with the camera settings that would be used during the experiment. After calibration, the fibers were individually picked up using tweezers from the substrate on which they had been printed and placed on the needle that had been used to inject the fibers into the settling chamber one at a time (more details in ref (30)). After a fiber was released in the settling chamber, an external trigger started recording in the synchronized cameras at 1400 or 2900 frames per second depending on the configuration. When a fiber was successfully observed by the cameras, the corresponding images were stored for further image postprocessing and data analysis.

#### 2.1.3 Verification of the Setup

To verify that the experimental setup can accurately observe the settling velocity of the fibers, we dropped spheres with different diameters, whose steady-state settling velocities are well-known, and compared them with the experimental settling velocities. A set of 185 μm (107 μm) diameter spheres was used for the 2 mm (1 mm) long fibers with an aspect ratio of 50. The sphere diameter was chosen such that its steady-state settling velocity is similar to that estimated by the model of Bagheri and Bonadonna (2016).^28^ We then compared our experimental settling velocities of the spheres with the empirical model of Clift and Gauvin (1971)^42^ and found a maximum relative difference of 4.7%. The average standard deviations of all the experiments were 2.1% (0.8%) for a 185 μm (107 μm) diameter sphere.

#### 2.1.4 Image Postprocessing

During image postprocessing, the centroid position (*cx*, *cy*) of a fiber was determined for each image based on a calibrated three-dimensional coordinate system developed from a three-dimensional calibration. The process of calibration and extracting the resulting images with centroid positions are described in detail in Bhowmick et al.^30^ The distance traveled by a particle between two frames was calculated as the hypotenuse of right triangles with base *cx*_*n*+1_ – *cx*_*n*_ and altitude *cy*_*n*+1_ – *cy*_*n*_, where subscripts *n* and *n* + 1 correspond to two subsequent images. The settling velocity was computed as this distance multiplied by the frame rate of the camera. In our experiments, fibers tended to decelerate (indicating that they were released at speeds faster than their terminal velocity) but reached a constant velocity within the field of view of the bottom cameras. A constant falling speed of a fiber over a wide range of video frames (the slope change of the regression line does not exceed 10%) indicates a successful experiment, and such velocities are counted as terminal velocities *w*_t_.

Noise in the measured particle velocity (caused by inaccuracies in the estimation of the centroid position of the particle projection) was filtered out by removing velocity values in the time series that deviated from the mean velocity by more than 3 standard deviations. This assumption was applied to each experimental postprocessed settling velocity time series, and then the settling velocities were averaged over the number of experiments for each fiber type.

### 2.2 Atmospheric Transport Model FLEXPART

We use a modified version of the Lagrangian dispersion model FLEXPART v10.4^43^ as a tool to investigate the effect of gravitational settling of particles of different sizes and geometries on atmospheric transport. The model simulates the transport, turbulent diffusion, convection, as well as dry and wet deposition of tracers both at global and local scales. For the calculation of the particles’ trajectories, FLEXPART uses the mean winds from the meteorological input data and parameterized stochastic turbulent motions in the turbulent atmospheric boundary layer. For gases, “particle” trajectories in FLEXPART represent the movement of air, but, to represent the movement of physical particles, particle trajectories can also include the vertical motion caused by gravitational settling. The calculated gravitational settling velocities are also used as inputs to the dry deposition scheme.

#### 2.2.1 Gravitational Settling Scheme

The trajectories and deposition of coarse-mode particles are influenced by their settling or terminal velocity *w*_t_. In FLEXPART, the settling velocity is determined at every time step and added to the vertical wind velocity.^43^ It is defined by Newton’s second law with an additional Cunningham slip correction factor *C*_cun_ (which is close to 1 for particles of the sizes considered here)
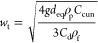
1where *g* is gravitational acceleration, *d*_eq_ is particle diameter, ρ_p_ and ρ_f_ are the densities of the particle and the fluid, respectively, and *C*_d_ is a drag coefficient, which is of the greatest interest since it is shape-dependent. The main challenge is to find the best mathematical solution for *C*_d_, considering nonspherical particle shapes.

#### 2.2.2 Drag Coefficient of Spherical Particles

We implemented the drag coefficient scheme developed by Clift and Gauvin (1971)^42^ in FLEXPART. This scheme is valid for subcritical Reynolds numbers and is within 6% of experimental measurements.^44^
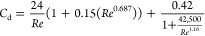
2

Comparisons with the settling velocities obtained in the experiments with spheres show that the scheme of Clift and Gauvin (1971) overestimates the settling velocity measured in our setup by 4.7% for particles of 185 μm in diameter and underestimates it by 0.9% for 107 μm in diameter particles.

#### 2.2.3 Drag Coefficient of Nonspherical Particles

Several empirical and semiempirical parameterization schemes describing the drag coefficient as a function of the particle shape have been developed.^28^ The recent study of Xiao et al.^45^ also provides a theory-based settling velocity model with a focus on cylindrical and flat fibers; however, it is strictly valid for small particles (i.e., *Re* ≪ 1) with extreme aspect ratios. According to Coyle et al.^46^ and Saxby et al.,^27^ the scheme of Bagheri and Bonadonna (2016)^28^ is considered one of the best-performing models when compared to experimental data. The model is suited for both regular and irregular particle shapes and is valid for a wide range of particle Reynolds numbers (up to *Re* = 3 × 10^5^). Bagheri and Bonadonna’s (2016)^28^ approach takes into account flatness (*f* = *S*/*I*) and elongation (*e* = *I*/*L*), where *L* is the longest, *I*—the intermediate, and *S*—the smallest dimension of the particle. Moreover, the scheme predicts the drag coefficients for random orientation, minimum projection area and maximum projection area orientation of a particle in liquids or gases by taking into account the particle-to-fluid density ratio. Maximum-drag orientation here means that the particle’s maximum projection area is normal to the particle’s relative velocity vector, which yields the maximum drag.

We implemented the simplified shape correction scheme of Bagheri and Bonadonna (2016)^28^ into the gravitational settling scheme of FLEXPART v10.4. The chain of equations in Table S1 (Supporting Information) describes the drag coefficient *C*_d_, which is a function of *Re* and Stokes’ *k*_S_ and Newton’s *k*_N_ drag corrections, for any geometric shape for random and maximum-drag orientations. The simplified version, in contrast to the full model, neglects the term *d*_eq_^3^/*LIS* in the calculation of Stokes’ shape descriptor, *F*_S_, and Newton’s shape descriptor, *F*_N_.

### 2.3 Atmospheric Transport of Fibers: Sensitivity Analysis

To study the impact of particle shape on particle transport in the global atmosphere, we performed transport model simulations using FLEXPART. The simulations used hourly ERA5 reanalysis of global meteorological data^47^ with 137 vertical levels and a grid resolution of 0.5 × 0.5°. To investigate the sensitivity of the model to the shape of the particles, we simulated the atmospheric transport of spheres and fibers with the same equivalent volume. We simulated fibers with a density of 1220 kg/m^3^, an equivalent diameter of 75 μm, and three different aspect ratios: 20, 50, and 100. For fibers with AR = 20, this results in a particle length of 483 μm and a diameter of 24 μm. For fibers with AR = 50 (AR = 100), these dimensions are 889 (1411) and 18 (14) μm, respectively. Several studies reported measured sizes in this range for microplastic particles deposited in remote regions.^16,18−20^ Since our experiments have shown that the observed settling velocities are best fitted by settling velocities calculated by averaging the drag coefficients for random and maximum-drag orientations (see Table 1), we chose the average drag coefficient for the FLEXPART simulations.

We performed two types of simulations. In the first type, we released particles at a height of 10–100 m above ground level from five different geographical locations, representing different climatic regions and circulation patterns: northern Italy (42°27′N 10°20′E), northwest Russia (62°00′N 53°00′E), Shanghai, China (31°20′N 121°50′E), Svalbard, Arctic (78°20′N 16°00′ E), and southwest Pacific (12°00′S 147°00′E) (Figure S3). Separate instantaneous releases of 10 000 particles were done once per day at local noon for a 1 year period. The model output consisted of three-dimensional atmospheric concentration fields and two-dimensional wet and dry deposition fields with a resolution of 0.5° and a nested grid with a resolution of 0.05°. Based on these simulations, we determined average travel distances and residence times in the atmosphere. The average horizontal transport distance *D* from the release point is given by
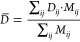
3where *D*_*ij*_ is the distance of grid cell *ij* from the release point and *M*_*ij*_ is the total deposited mass in grid cell *ij*. Residence times were determined as e-folding times *t*_e_ from the relative decrease in the total particle mass in the atmosphere as a function of time. The mean values over the number of releases for the relative mass decrease were fitted to the exponential function *y* = e^–*bx*^. Then the e-folding time *t*_e_ was defined as the inverse coefficient *b*.

For the second type of FLEXPART simulation, we released particles globally according to an estimate of the distribution of microplastic emissions. In an inverse modeling study, it was estimated that on average 0.7 *T*_g_ of microplastic particles are emitted annually from land-based sources.^48^ Assuming that the microplastic emissions are closely related to human activities, we used gridded global population density data^49^ as a proxy to spatially disaggregate the global emissions with a resolution of 1 × 1°. 1.4 million particles were released in FLEXPART according to this emission distribution. We simulated the atmospheric transport and deposition for 14 months (November 2017 to December 2018), of which the first two months were considered model spin-ups and discarded. While the uncertainties in global microplastic emissions are very high,^48,50−52^ we consider this simulation a realistic approximation of global atmospheric microplastic transport for our purpose of exploring the sensitivity of the simulated transport to the particle shape. Four simulations were performed, in which it was assumed that all microplastic emissions are either spheres with a diameter of 75 μm or cylinders of the same volume with AR values of 20, 50, and 100.

Aerosols are affected not only by dry deposition but also by wet deposition. For instance, experiments suggest that microplastic particles can affect cloud formation.^53^ For both types of simulations, we first assumed that the simulated microplastic particles are hydrophobic and chemically inert, which corresponds to relatively low in-cloud scavenging efficiencies. Consequently, following Grythe et al.,^65^ we chose their in-cloud scavenging efficiencies for cloud condensation nuclei (CCN) and ice nuclei (IN) as 0.001 and 0.01, respectively. However, for the second type of simulations, we consider that photo-oxidation can alter microplastic hydrophobicity^54^ and also performed sensitivity studies assuming that the particles are hydrophilic, setting their CCN and IN efficiencies to values of 0.5 and 0.8, respectively.

## 3 Results and Discussion

### 3.1 Settling Dynamics of Fibers

Examples of superimposed camera snapshots of fibers settling through the lower region of the field of view of the bottom cameras, where the fibers had already reached a terminal velocity *w*_t_, are shown in Figure 1b,e, clearly revealing the fibers’ position and orientation. The entire camera recordings of the settling behavior are shown in Figure S4. Besides the settling velocities of the fibers, we also studied their orientation dynamics (Figures 1d,g, S4, and S5b,d). We find in similitude with previous work^30,31,33^ that fiber-like particles achieve a terminal steady-state orientation with their maximum projection area perpendicular to the settling direction. Beyond the existing knowledge, we further observe that depending on their aspect ratio, fibers can approach their steady-state orientation with or without oscillations, which is in agreement with the theoretical predictions about oscillations in Figure 4 of Bhowmick et al.^30^

In particular, the fiber oscillations decrease as the fiber diameter decreases (Figure S4). For example, regardless of their shape, the fibers with 1 mm length and AR = 20 had reached both their steady-state orientation, which is 90°, and settling velocity in the section which was observed by the bottom cameras (Figures 1b–d and S5a,b). Their oscillations in orientation were already dampened as they fell through the section observed by the top cameras (Figure S4a–c). In contrast, the fibers with 2 mm length and AR = 20 did not reach a steady-state orientation for the entire duration of the camera recording, but they had nevertheless reached a terminal velocity when observed by the bottom cameras (Figures 1e–g, S4e–g, and S5c,d). Our experiments indicate that the fiber oscillations decay exponentially in air, with a decay constant of the order of 10 ms. From the results shown in Figure 1, one crucial observation can be deduced: variations in particle orientation have no discernible influence on the terminal velocity. This is because the orientation of the particles can continue to oscillate around the steady-state orientation after the fibers have reached their terminal velocity. For the terminal velocity to be affected by a change in particle orientation, a particular orientation must be sustained long enough, i.e., comparable to or longer than the response time of the particle to relax to its steady-state velocity due to drag. This time scale for the Stokes flow (particle Reynolds number less than unity) is called the Stokes response time τ_p_ = (1/18)(ρ_p_/ρ_f_)*Ld*_cyl_/ν,^30^ which for the fibers ranges from 46 to 733 ms, where ρ_f_ and ν are, respectively, the density and kinematic viscosity of air.

In the atmosphere, the transport of the microplastics is affected by turbulence characterized by the dissipation rate of turbulent kinetic energy, ε, which typically varies from 1 × 10^–2^ to 1 × 10^–4^ m^2^ s^–3^.^55,56^ The smallest vortices in such a turbulent atmosphere, called Kolmogorov microscales, range in size  from 0.8 to 2.4 mm, with time scales for vortex turnover, τ_η_ = (ν/ε)^1/2^ ranging from 40 to 400 ms.^56^ Therefore, our experimental fibers are of the same size as the Kolmogorov microscale, but the time scales for alignment in fiber orientation are smaller than the smallest time scales typically encountered in atmospheric turbulence. Furthermore, the Kolmogorov velocity scale, *u*_η_ = (νε)^1/4^ varying from 6 × 10^–3^ to 2 × 10^–2^ m s^–1^, is smaller than the settling velocities of all experimental fibers. An important insight of these new findings is that the settling velocities obtained from our experiments in still air are representative also of the settling velocities of fibers in the turbulent atmosphere. Consequently, this finding allows us to constrain gravitational settling in atmospheric transport models in combination with our laboratory data.

**Figure 2:**
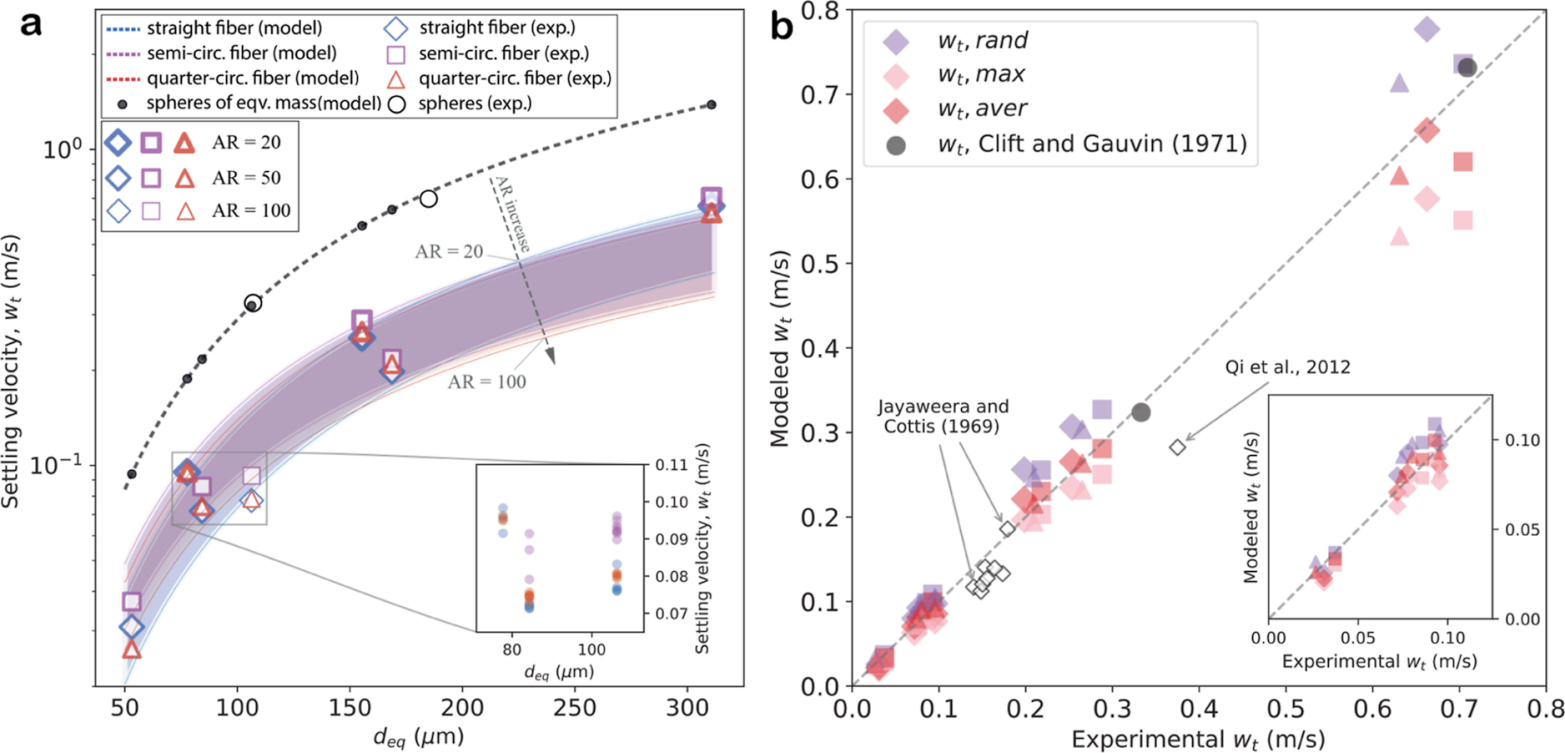
Observed and modeled gravitational settling velocities. (a) Settling velocities as a function of particle size, expressed as the diameter of a sphere of equivalent volume. Modeled values using the shape correction scheme are shown as a black dashed line for spheres and as colored shaded areas covering the range of aspect ratios from 20 to 100 for straight (blue), semicircular (purple), and quarter circular (red) fibers. Experimental mean values for each shape are represented by open markers, coded by color for shape type and by thickness for aspect ratio. The spread in the results of repeated experiments for individual particle types is smaller than the size of the symbols. The inset gives an example of the experimental data spread for a limited size range on a linear velocity scale. Black dots show the calculated settling velocities of spheres. (b) Scatter plot of observed and modeled settling velocities. Modeled values were calculated for random (purple), maximum-drag (pink), and averaged (red) orientations. Black open rhombuses show previously published experimental results by Qi et al.^33^ and Jayaweera and Cottis (1969).^32^

Our experimental results reveal that the measured terminal velocities for straight fibers and semicircular and quarter circular fibers are only 24–51% of the velocities of spheres with the same volume (Figure 2a and Table 1). The largest differences are found for the particles with the largest aspect ratios (AR = 100), for which the settling velocities are less than one-third of those for spheres of the same volume. For a given volume and aspect ratio, the differences between the velocities of straight, semicircular, and quarter circular fibers are relatively small (on average, 12% with a maximum deviation of ∼26% for particles of size 1000 × 10 μm). Nevertheless, straight fibers have the lowest settling velocities, and semicircular fibers have the highest settling velocities. On average, straight fibers settle with 38% (minimum value of 24%) and semicircular fibers with 41% (minimum value of 29%) of the velocity of spheres of the same volume.

Recently, the model of Bagheri and Bonadonna (2016)^28^ was shown to be associated with small errors in estimating the settling velocity of microplastics of various shapes in water.^46^ The model can be fitted to predict the settling velocity for nonspherical particles when they fall with their minimum, maximum, or random projection areas facing downward, and it is also applicable to settling in air. Figure 2b compares this model with the observations. With its maximum drag orientation configuration, the model agrees well with the measurements (Pearson correlation coefficient *r* = 99.8, *p*-value = 2 × 10^–7^) but, on average, systematically underestimates the observed velocities by 13.3 ± 10.0% for straight fibers. For random orientation, the model has slightly higher deviations with respect to the measurements and systematically overestimates them by 16.8 ± 8.4% (Pearson correlation coefficient *r* = 99.9, *p*-value = 8 × 10^–8^). The better agreement between the maximum projection model and the measurements is consistent with the observation that particles mostly stabilize with their maximum projection area facing downward (Figures 1, S4, and S5). However, even better agreement is found for the averaged orientation (Pearson correlation coefficient *r* = 99.9, *p*-value = 2 × 10^–7^), which is the arithmetic mean of the predictions for the maximum and random orientations and allows for some oscillation around the maximum drag orientation. With the average-orientation model, no systematic bias occurs, and the deviations are further reduced to 8.6 ± 8.2%. The results for semicircular and quarter circular fibers are similar (Table S2).

### 3.2 Simulation of Atmospheric Dispersion of Microplastic Fibers

The excellent agreement between the observed and modeled settling velocities within less than 10% suggests that we can use the Bagheri and Bonadonna (2016)^28^ scheme with averaged orientation to realistically simulate gravitational settling in the atmosphere. To explore the effects of the shape dependence of gravitational settling on the global transport of microplastics in the atmosphere, we therefore implemented this scheme into the Lagrangian atmospheric transport model, FLEXPART^43^ (Table S1).

**Figure 3:**
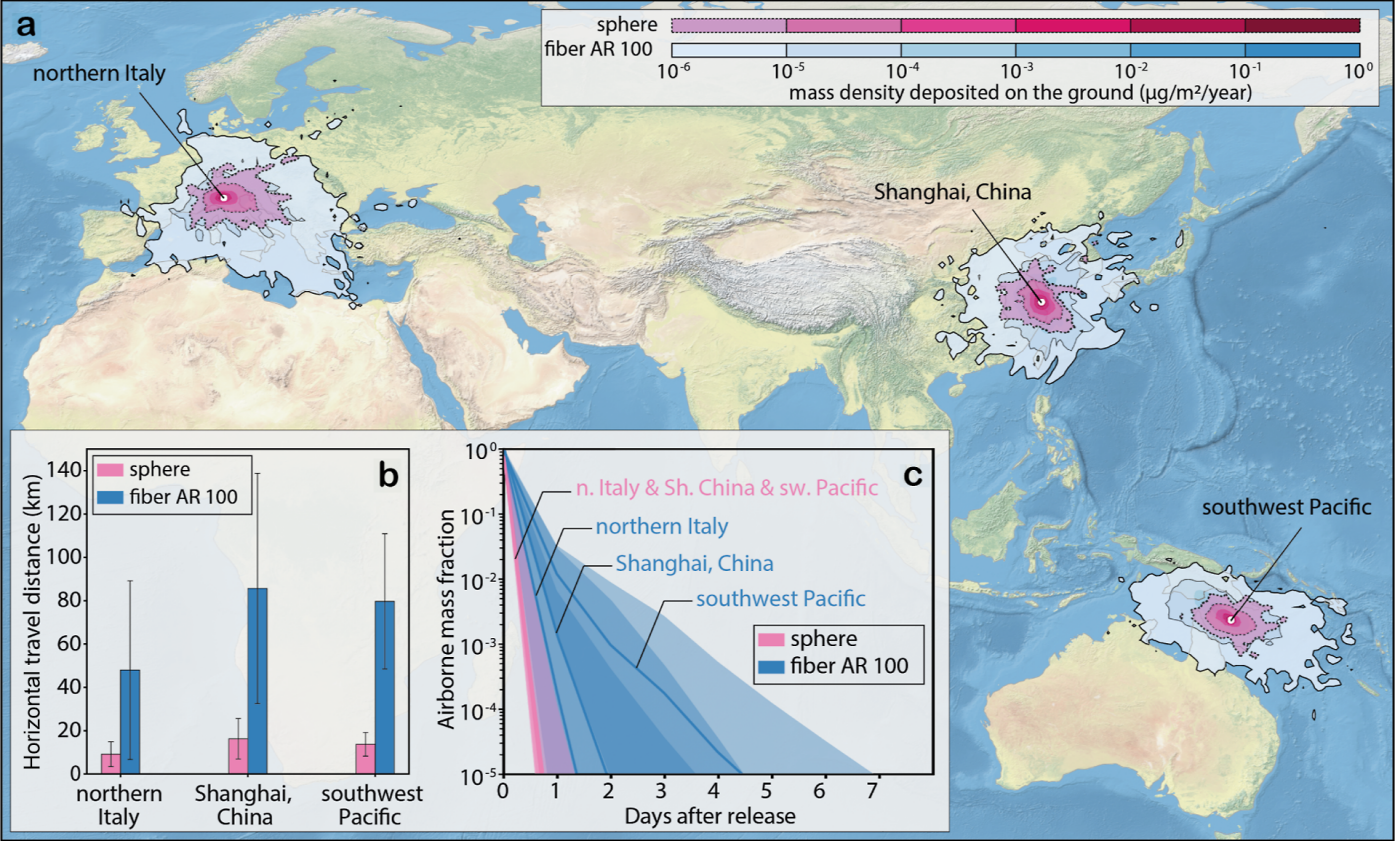
Characteristics of microplastic transport in the atmosphere. Shown are FLEXPART model simulation results for three release points and for microplastic particles with an equivalent diameter of 75 μm for spheres (pink) and straight fibers with an aspect ratio of 100 (blue). (a) Annual mean total deposition from the atmosphere for spheres and fibers. The values of the mass density are limited to 10^–6^ μg/m^3^/year, and the full range of values is shown in Figure S7. (b) Annual mean values of the horizontal transport distances (colored bars) and their standard deviation (whiskers) for spheres and fibers released from the three points. (c) Decrease of the atmospheric microplastic burden as a function of time after release of spheres and fibers from the three points, with the solid lines showing the median values and the shading indicating the range between the 25th and 75th percentiles.

The deposition patterns resulting from the first set of simulations clearly show that the atmospheric transport distances depend systematically on the particle shape (Figures 3 and S6). For instance, for a release point in northern Italy, all deposition occurs virtually in central and southern Europe for spheres, whereas straight fibers with AR = 100 are also deposited in northern Africa and northern Europe and small amounts even reach the Arctic (Figure S7a–d).

On average, across our five release points, the mean atmospheric transport distances of fibers with aspect ratios of 20, 50, and 100 are, respectively, 157 ± 26, 272 ± 50, and 394 ± 79% greater than those for spheres with the same volume (Figure S6). This is a result of the longer residence time of fibers in the atmosphere compared to that of spheres, with e-folding times of 2.4, 4.1, 4.6, and 5.3 h for spheres and straight fibers with aspect ratios of 20, 50, and 100, respectively. Consequently, the median fractions of their emitted mass residing in the atmosphere differ by orders of magnitude after a few days (Figures 3c and S8). The spread in the mass fraction frequency distributions is also broader for fibers than for spheres, indicating a strongly enhanced probability for very long-range transport. This explains why deposition and mass concentration fields extend over much larger regions for fibers than for spheres (Figures 3a, S7, and S9).

**Figure 4:**
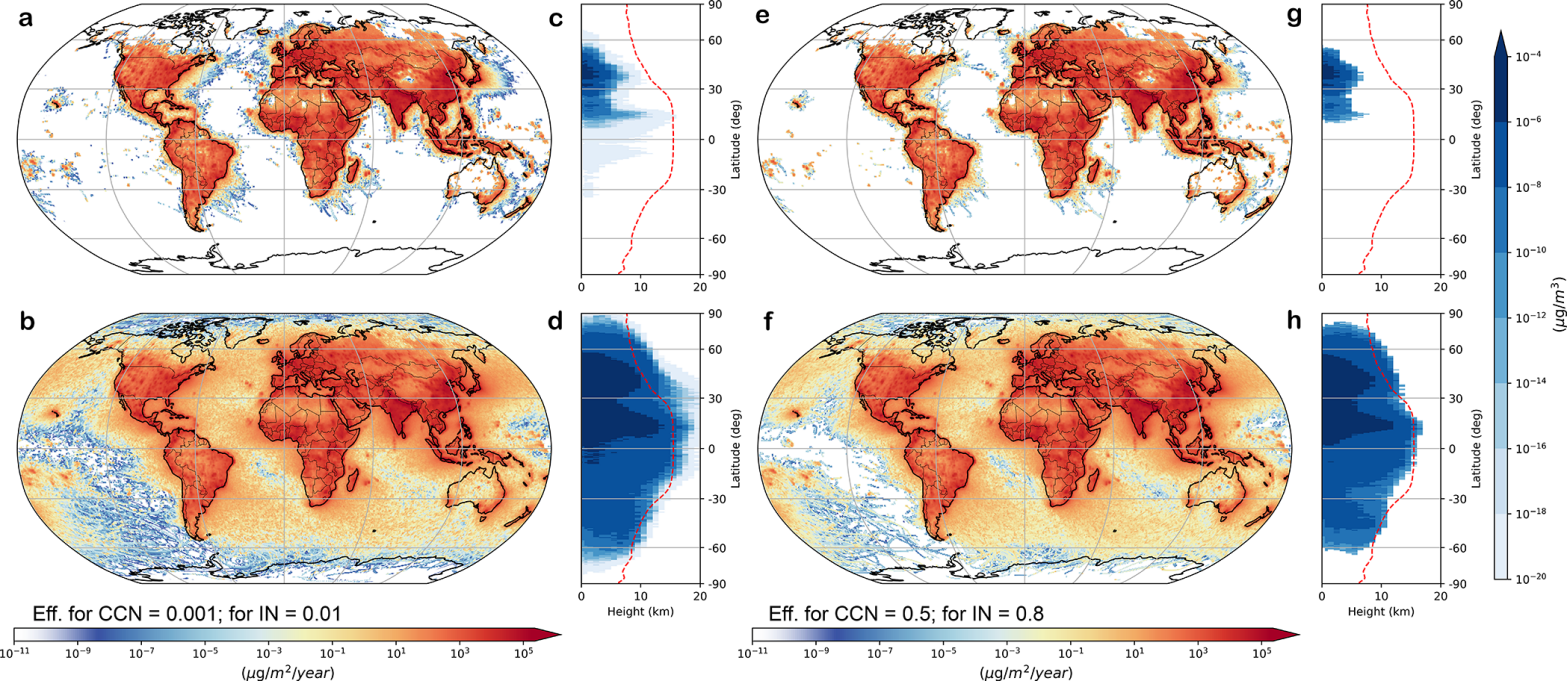
Shape dependence of global microplastic deposition and vertical distribution in the atmosphere. Shown are results for particles with an equivalent diameter of 75 μm that only deviate in their shape, for (a,e) spheres and (b,f) straight fibers with an aspect ratio of 100 and hygroscopicity: hydrophobic (a–d) and hydrophilic (e–h) microplastics. Zonal median atmospheric mass concentration of microplastics as a function of latitude and altitude for (c,g) spheres and (d,h) straight fibers with an aspect ratio of 100. The dashed red line indicates the tropopause height, extracted from ERA5 reanalysis data.^47^

The second set of simulations shows that the deposition of spheres is strongly focused on the densely populated source regions, while fibers are deposited globally (Figures 4a,b,e,f and S10a–d,i–l). For the fibers with AR = 100, only remote regions in the south Pacific and interior Antarctica remain nearly unaffected by the deposition of both hydrophobic and hydrophilic microplastic particles.

For example, our simulations show a total deposition of 2.4 t/year (1.6 t/year) in the high Arctic (north of 75°N) for hydrophobic (hydrophilic) fibers with AR = 100, while volume equivalent spheres do not reach this region at all (Table S3). Our results explain why the high Arctic, despite its remoteness, has been reported to be heavily affected by microplastic deposition from the atmosphere,^20,57^ and why most of the microplastic particles found there appear to be fibers.^57^

Several studies discuss the impact of microplastics on the oceans^52,58,59^ and provide field evidence of their long-range atmospheric transport.^60^ Despite these advancements, the contribution of atmospheric transport to the contamination of microplastics in oceans remains highly uncertain.^4^ Our simulations with hydrophobic microplastics show that if all particles are spherical, 16 kt/year are deposited in the oceans, whereas for straight fibers with aspect ratios of 20, 50, and 100, the corresponding numbers are 18, 20, and 23 kt/year, respectively (Table S3). The same pattern with slight deviations is also observed for hydrophilic particles (Table S3). Furthermore, spheres are deposited mainly in coastal regions close to population centers, whereas the straight fibers also reach remote ocean areas. For the remote central Indian Ocean (Figure S3), ∼7 (∼2) times more mass of hydrophobic (hydrophilic) microplastics would be deposited if particles are shaped as fibers with AR = 100 than if they are spheres (Table S3).

Remote land regions are also contaminated more severely for fibers than for spheres, as shown in Figure 4a,b,e,f. For instance, the interior of Australia (Figure S3) receives 90% (50%) more hydrophobic (hydrophilic) microplastic mass for fibers with AR = 100 than for spheres. These results are in good agreement with the fact that in relatively remote regions, such as national parks, mainly microplastic fibers and other nonspherical particles have been reported,^15−20,60^ whereas reports of microplastic spheres are less common.

The simulated vertical distribution of microplastic particles in the atmosphere is also very different for spheres and straight fibers (Figure 4c,d,g,h), with the fibers reaching altitudes much higher than those of spheres. For instance, the mass of hydrophobic (hydrophilic) microplastics at altitudes greater than 4 km above the surface is 5.5, 9.0, and 13.3 (3.4, 5.2, and 7.4) times higher for straight fibers with AR = 20, 50, and 100, respectively, than for spheres (Figure S10e–h,m–p). The effective transport of fibers to high altitudes could have substantial implications for ice cloud formation since microplastic particles are thought to serve as ice nuclei.^53^ The larger surface area of fibers compared to that of spheres of the same volume, i.e., lower sphericity, is another important factor, possibly making microplastic fibers highly effective ice nuclei with an impact on climate.^54^

It seems even more likely that microplastic fibers of smaller sizes can reach the stratosphere with potentially severe consequences for the ozone layer. For example, for fibers with lengths of 94 and 75 μm and AR = 100 (corresponding to spheres of equivalent volume with a diameter of 5 and 4 μm) with respective densities of 900 kg m^–3^, typical of polyurethane, and 1400 kg m^–3^, typical of polyvinyl chloride, we obtain settling velocities of less than 0.2 mm/s at stratospheric altitudes between 20 and 30 km. This is slower than the minimum mean upwelling velocity of the Brewer–Dobson circulation near the tropical tropopause,^61,62^ leading to the fibers’ ascent into the stratosphere, where cloud removal processes are not effective.

Many plastic products contain bromine or chlorine, for instance, as flame retardants,^63^ and polyvinyl chloride consists of more than 50% chlorine by weight. Plastics degrade under exposure to ultraviolet (UV) light and can release halogen-containing gases.^64^ Their large surface area compared to their volume, the horizontal orientation of their maximum projection area, and long residence times of possibly years make a complete disintegration of halogen-containing fibers exposed to the extreme UV levels and high ozone concentrations in the stratosphere plausible. The released bromine and chlorine compounds could participate in the catalytic destruction of ozone, similar to those released from chlorofluorocarbons and halons regulated by the Montreal Protocol. It is important to note that the amount of microplastic potentially reaching the stratosphere is extremely sensitive to the size distribution of the emitted microplastic particles. However, currently, both the shape and size distribution of nano- and microplastic particles and even their total global emissions are largely unknown, and more research is needed for their characterization. At this point, we emphasize that the specific fiber shape of many microplastic particles is important for their potential transport into and in the stratosphere.

We have shown that the shape of microplastics is an important factor for their global presence in the environment: the more nonspherical their shape, the larger their horizontal and vertical transport range. Our findings demonstrate that microplastics can be transported in the atmosphere to almost any point of the globe and are present throughout the troposphere and possibly the stratosphere, when considering both low and high in-cloud scavenging efficiencies. Novel laboratory experiments and model analyses carried out here diminish uncertainties regarding the settling behavior of fibers in the atmosphere, leading to more accurate model simulations of the atmospheric concentration and deposition patterns. The slow settling velocities obtained for fibers also make it seem plausible that microplastic fibers can reach the stratosphere, where they might endanger the ozone layer. Moreover, particles of other shapes than straight or bent fibers, such as films^17,18^ or particles with nonsmooth surface textures, may have an even larger atmospheric transport potential.

## Data Availability

The data underlying this study are openly available in Zenodo at https://doi.org/10.5281/zenodo.10391708.

## Supporting Information Available

Photographs of the experimental setup and printed particles, additional snapshots of fiber settling, time series of the particle’s vertical velocity and orientation angle, particle residence time, horizontal displacement, deposition and mass concentration fields of all release points, extended figure of global microplastic deposition and vertical profile of their mass concentrations, table with equations to calculate the drag coefficient for three types of orientation, and data on the deposited mass of microplastics in remote regions (PDF)
