# Supplementary material for: Shape Matters: Long-Range Transport of Microplastic Fibers in the Atmosphere
Source: Environ Sci Technol. 2023 Dec 27;58(1):671–82. doi: 10.1021/acs.est.3c08209 (PMC10785798; doi:10.1021/acs.est.3c08209)
Supplement: Supplementary file 1 — es3c08209_si_001.pdf [file es3c08209_si_001.pdf]

Shape matters: long-range transport of microplastic fibers in the atmosphere

Daria Tatsii,[*,†] Silvia Bucci,[†] Taraprasad Bhowmick,[‡,¶] Johannes Guettler,[‡] Lucie Bakels,[†] Gholamhossein Bagheri,[*,‡,§] and Andreas Stohl[†,§]

†*Department of Meteorology and Geophysics, University of Vienna, Universitätsring 1, 1010, Vienna, Austria*

‡*Laboratory for Fluid Physics, Pattern Formation and Biocomplexity, Max Planck Institute for Dynamics and Self-Organisation, Am Faßberg 17, 37077, Göttingen, Germany*

¶*Institute for the Dynamics of Complex Systems, University of Göttingen, Friedrich-Hund-Platz 1, 37077, Göttingen, Germany*

§*G.B. and A.S. contributed equally to this paper.*

E-mail: daria.tatsii@univie.ac.at; gholamhossein.bagheri@ds.mpg.de

Number of pages: 12

Number of Figures: 10

Number of Tables: 3

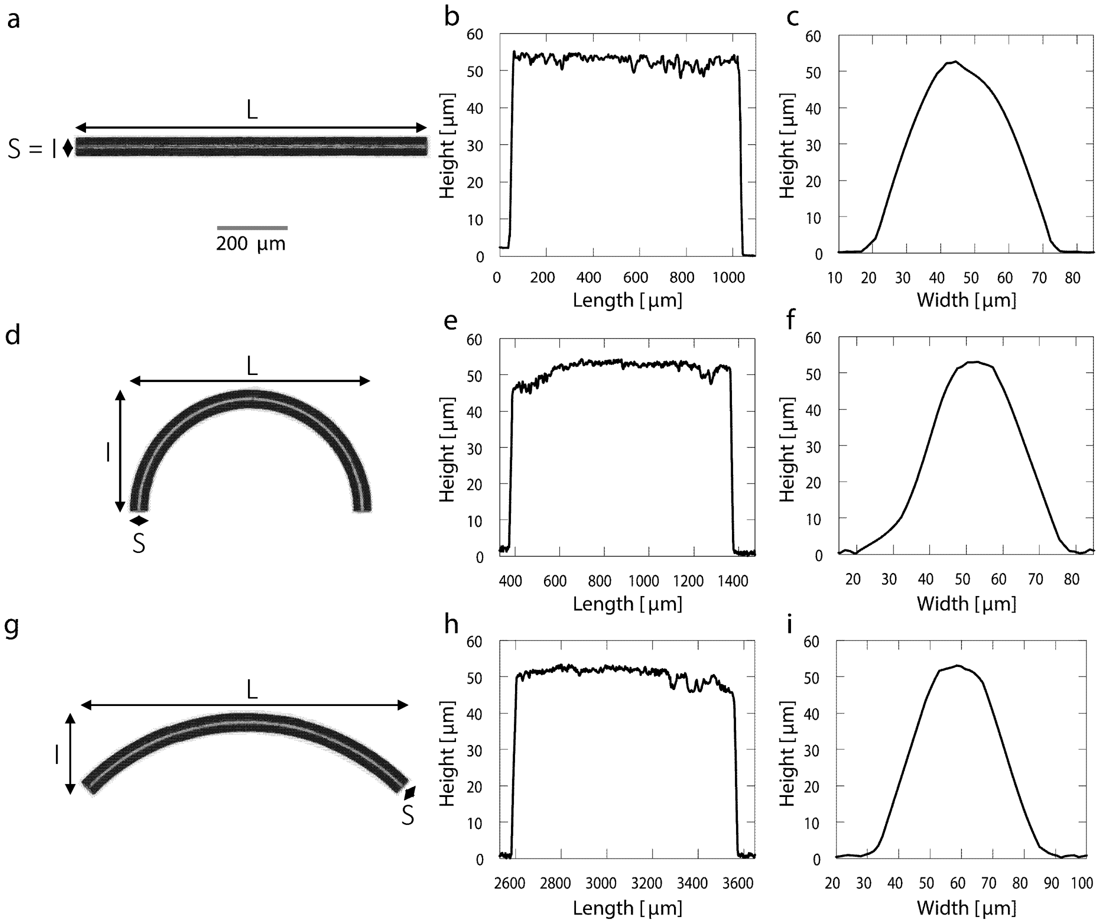

Figure S 1: **Images of the printed fibers obtained with 3D microscopic scans** with indication of longest L, intermediate I, and smallest S dimensions (panels in the left), and cross-sectional area along their actual length (panels in the middle) and width, i.e., diameter of the cylinder d_{cyl} (panels in the right) for (**a-c**) straight, (**d-f**) semicircular, and (**g-i**) quarter circular fibers of 1 mm length and 50 µm diameter.

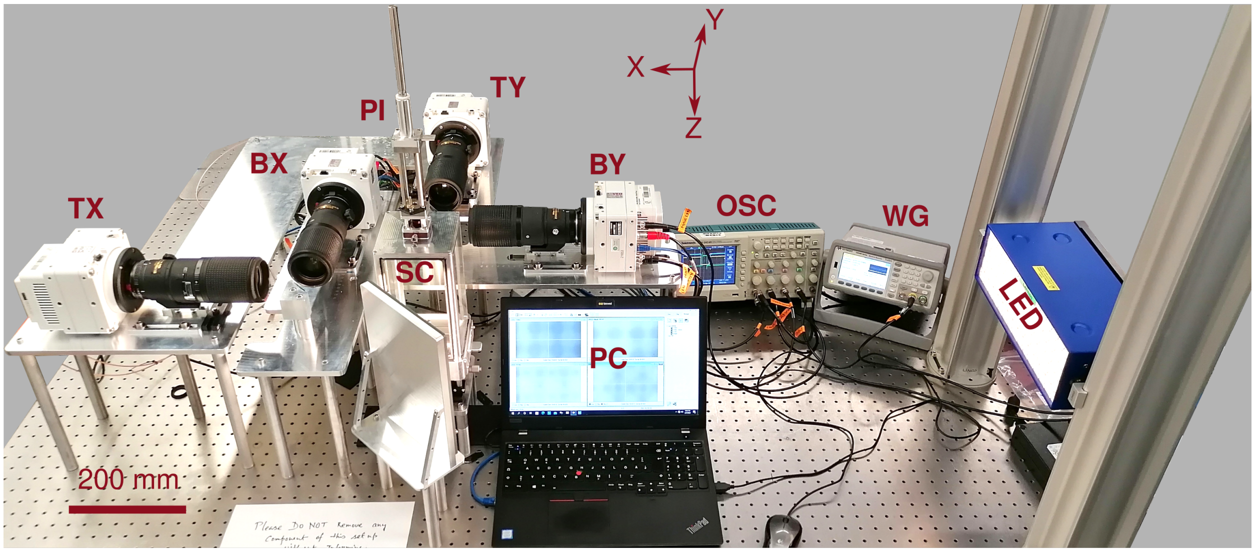

Figure S 2: **Experimental setup.** Photograph of the experimental setup of *the Göttingen turret*, which consists of two top cameras (TX and TY), and two bottom cameras (BX and BY) aligned in $X-$ and $Y-$ directions, the settling chamber (SC) where the fibers were released using the particle injector (PI) to let them settle under gravity ($Z-$ direction) in still air. Other components are oscilloscope (OSC), personal computer (PC), waveform generator (WG), and an pulsed LED unit (LED).

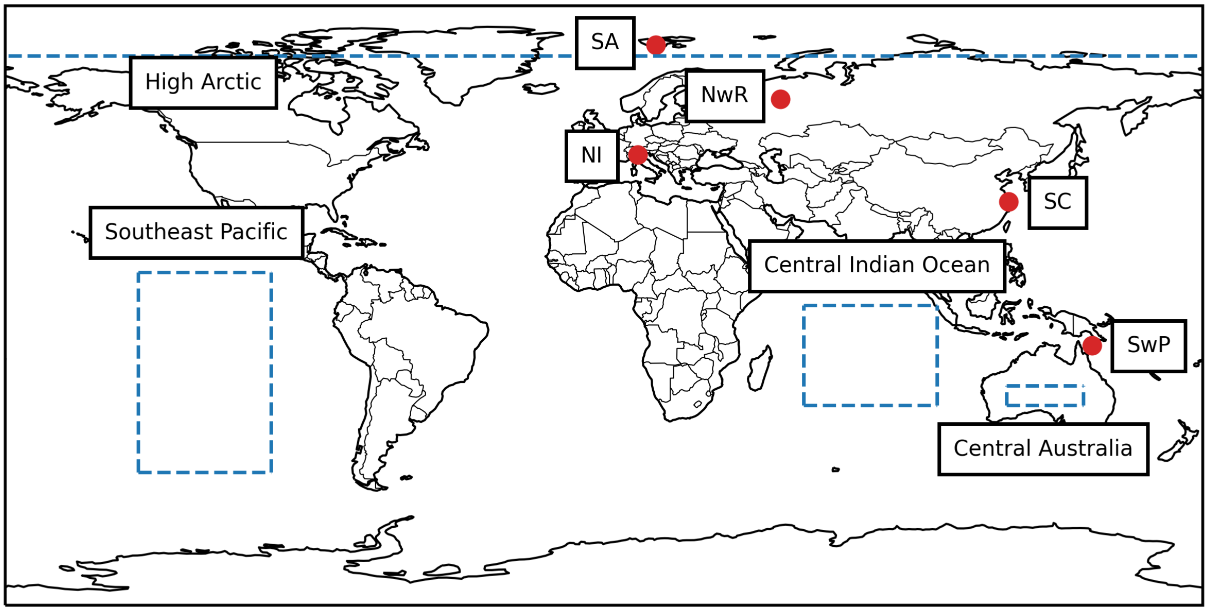

Figure S 3: **Map with release points (red dots) and selected remote regions for which we compared the microplastics deposition for different shapes (blue dashed contours).** Abbreviation SA stands for Swalbard, Arctic, NI - Northern Italy, NwR - Northwest Russia, SC - Shanghai, China, SwP - Southwest Pacific.

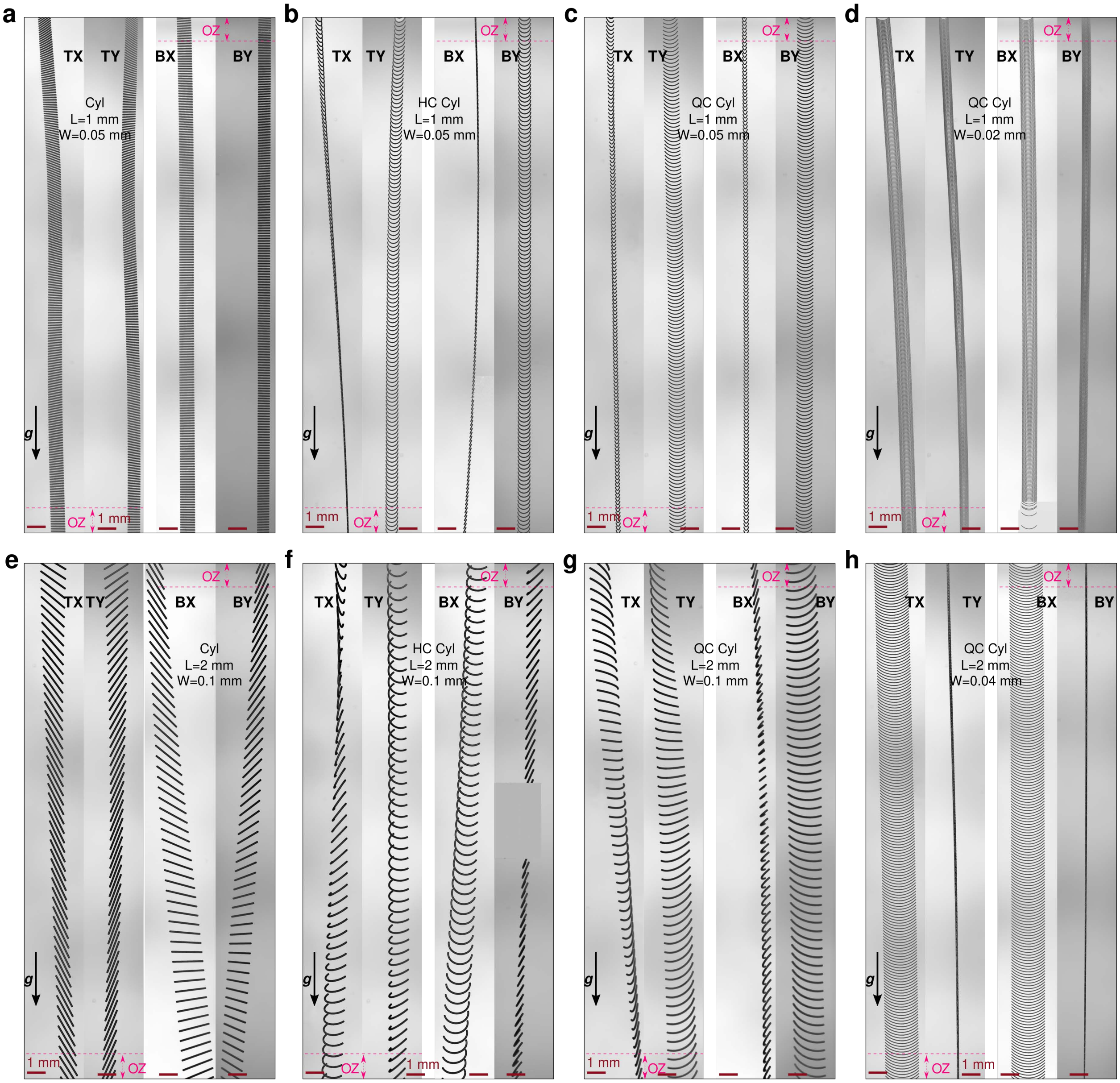

Figure S 4: **Snapshots of settling fibers of** 1 mm **(a-d) and** 2 mm **(e-h) length.** Straight (**a,e**), semicircular (**b,f**), quarter circular (**c,g**) cylindrical fibers, each of aspect ratio AR = 20; and quarter circular cylindrical fibers, each of AR = 50 (**d,h**). For each fiber, data are shown from the top cameras (TX and TY), and bottom cameras (BX and BY) for the entire vertical length of the camera observation volume.'OZ' refers to the overlapping zone between the top and bottom camera pairs, where the particle is observed by all cameras. Here L is the actual length of the particle and W is a width, i.e. diameter of the cylinder d_{cyl}.

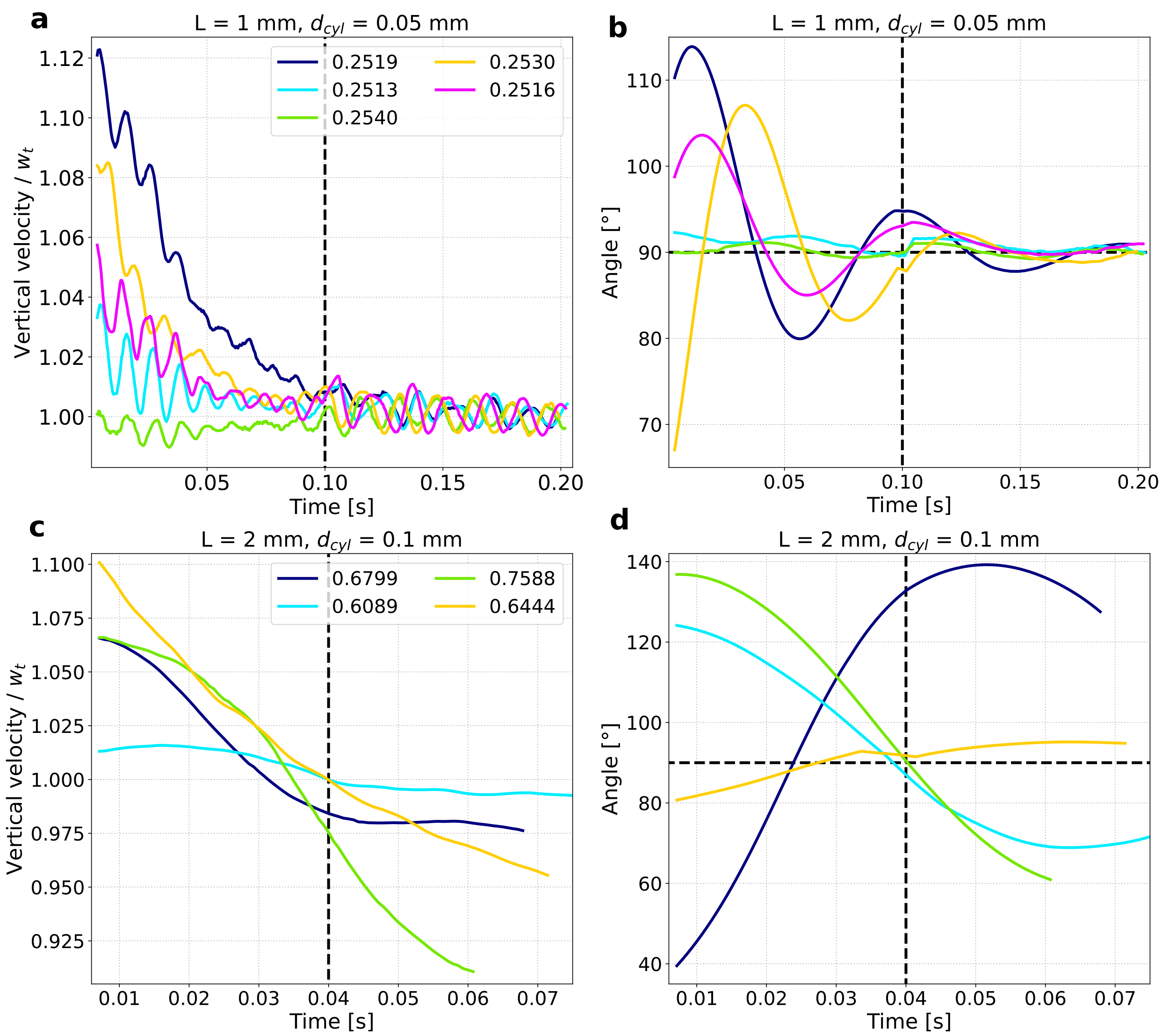

Figure S 5: **Time series of the normalized vertical velocity (a,c) and fiber orientation (b,d)** from experiments with straight cylinders of AR = 20. Top and bottom panels exhibit the measurements of fibers with length, L of 1 mm and 2 mm respectively, with corresponding fiber diameter, d_{cyl} of 0.05 mm and 0.1 mm. The half of the plots left to the vertical dashed line corresponds to post-processed data from the particle images captured by the top cameras (TX or TY), while the right half is captured by the bottom cameras (BX or BY). The vertical velocity is normalized by settling velocity, w_t for each experiment which is the average velocity observed by the bottom cameras, and is shown in the legends of panels (**a,c**) in unit of m s^{-1}. The horizontal dashed line in panels (**b,d**) corresponds to the steady-state orientation of the fibers of 90°.

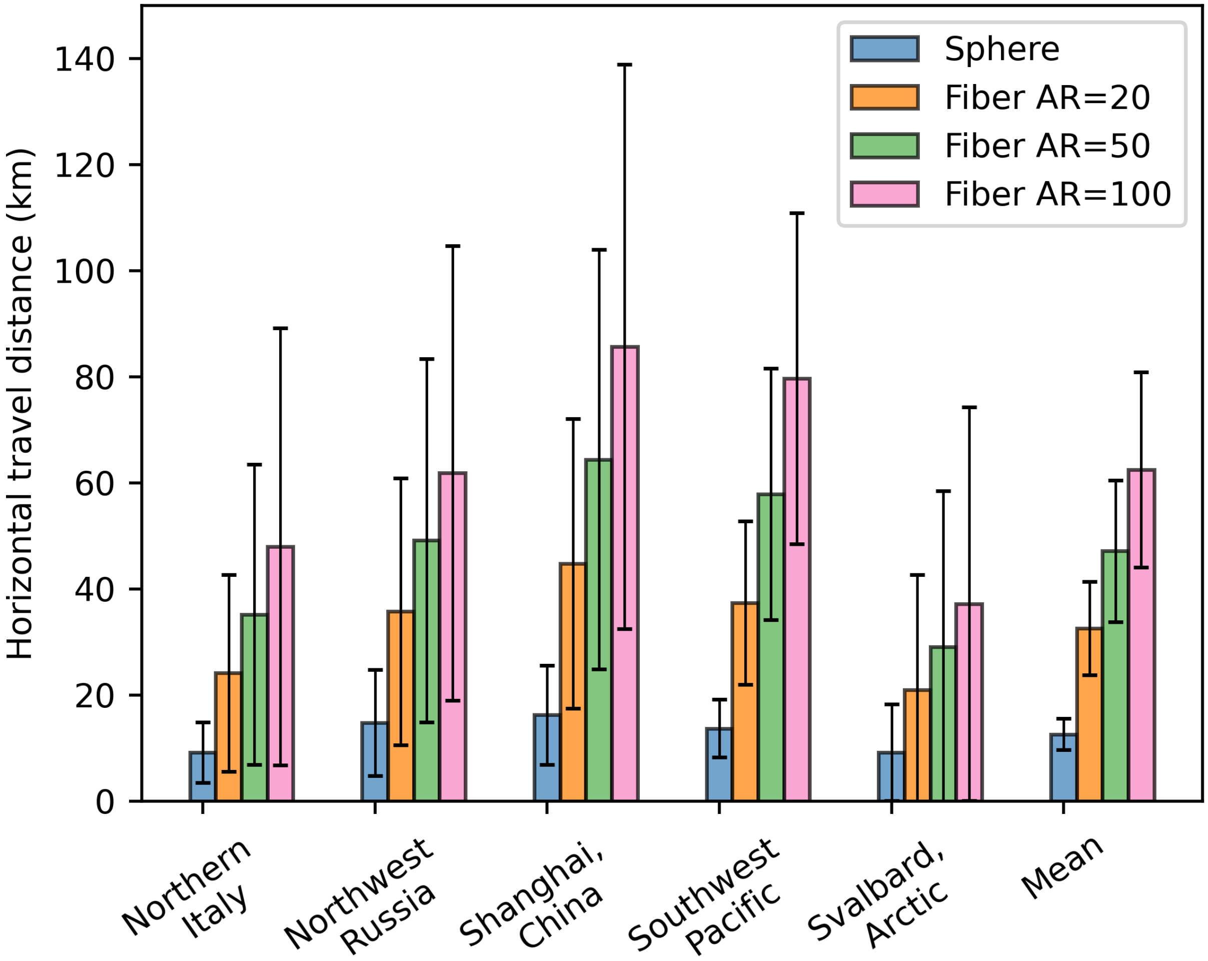

Figure S 6: **Horizontal transport distance of microplastic particles**. Shown are the annual mean values (colored bars) and standard deviation (whiskers) of transport distances for spheres and straight cylindric fibers of different aspect ratios with identical volumes for five different release points and their average.

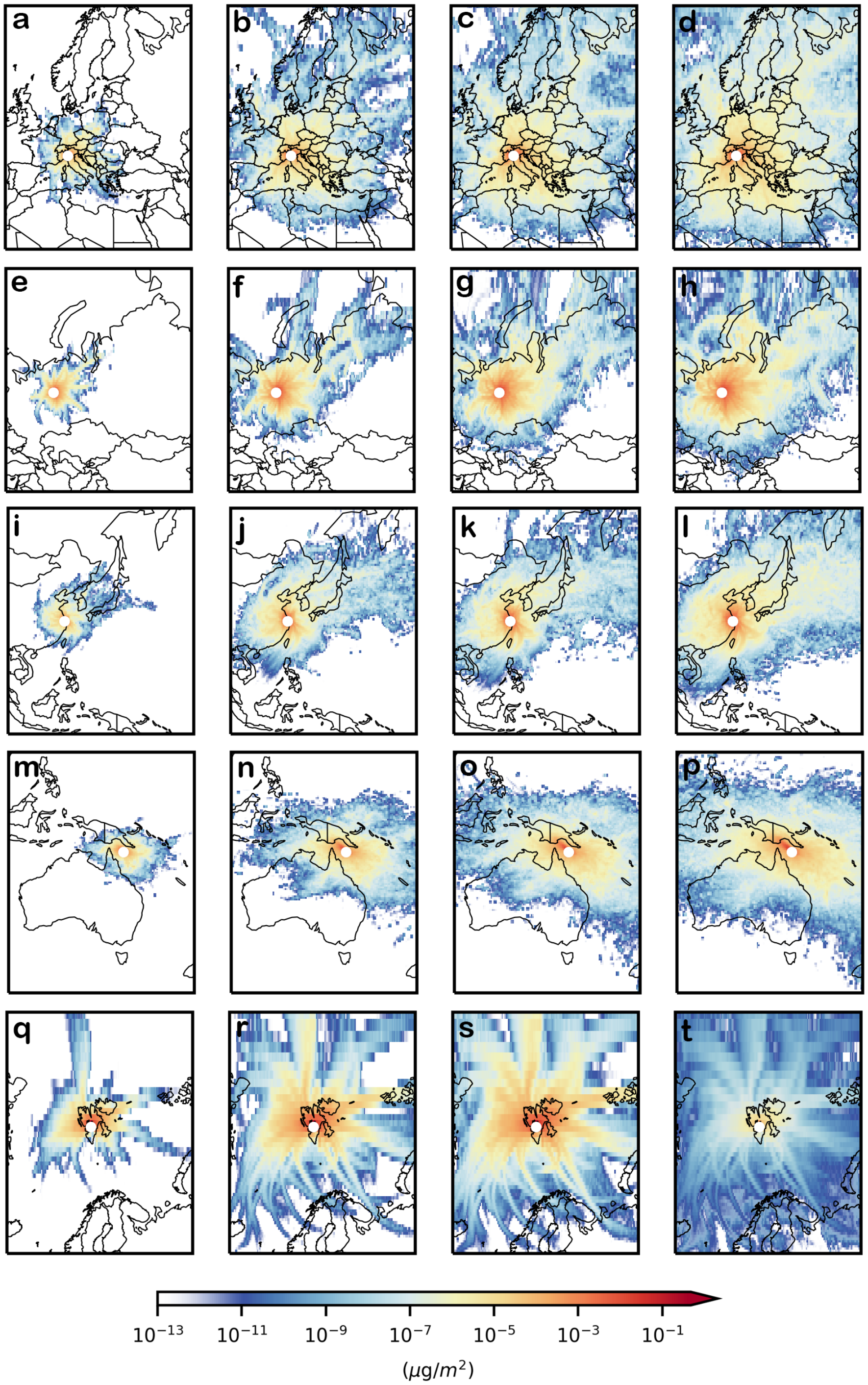

Figure S 7: **Annual microplastic deposition based on the emissions at 5 different points.** Shown are the annual deposition amounts for microplastic spheres (a,e,i,m,q) and straight cylindric fibers of different aspect ratios AR (AR=20 (b,f,j,n,r); AR=50 (c,g,k,o,s); AR=100 (d,h,l,p,t)) with identical volumes. The white dots mark the release points. Results are shown for release points in (**a-d**) Northern Italy, (**e-h**) Northwest Russia, (**i-l**) Shanghai, China, (**m-p**) Southwest Pacific, near Papua New Guinea, and (**q-t**) Svalbard, Arctic.

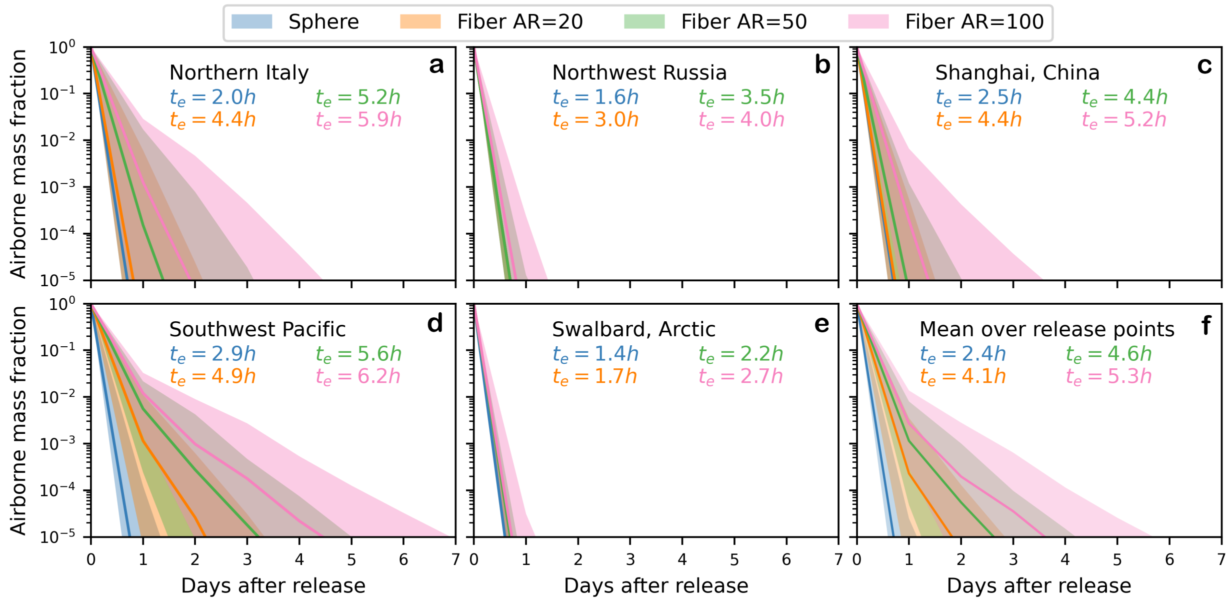

Figure S 8: **Decrease of the atmospheric microplastic burden for spheres and straight cylindric fibers of different aspect ratios with identical volumes as a function of time after the release**. The solid lines showing the median values and the shading indicating the range between the 25th and 75th percentiles. Results are shown for release points in (**a**) Northern Italy, (**b**) Northwest Russia, (**c**) Shanghai, China, (**d**) Southwest Pacific, near Papua New Guinea, and (**e**) Svalbard, Arctic. (**f**) Median values and uncertainties averaged over release points. Resulting e-folding residence times in the atmosphere are reported in each panel.

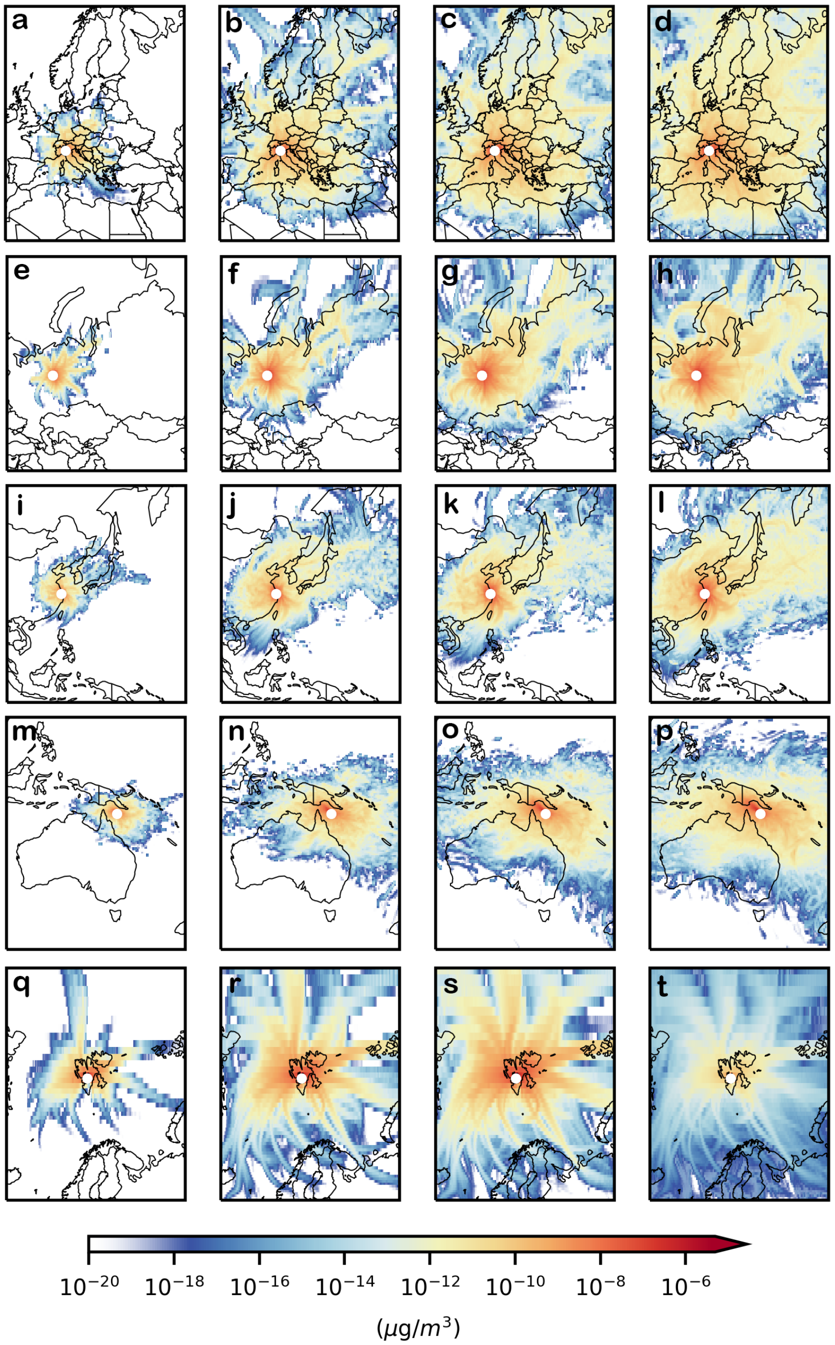

Figure S 9: **Annual mean mass concentration between the surface and 1000 m of height based on the emissions at 5 different points.** Shown are the annual mean concentrations for microplastic spheres and straight cylindric fibers of different aspect ratios (a,e,i,m,q) and straight cylindric fibers of different aspect ratios AR (AR=20 (b,f,j,n,r); AR=50 (c,g,k,o,s); AR=100 (d,h,l,p,t)) with identical volumes. The white dots mark the release points. Results are shown for release points in (**a-d**) Northern Italy, (**e-h**) Northwest Russia, (**i-l**) Shanghai, China, (**m-p**) Southwest Pacific, near Papua New Guinea, and (**q-t**) Svalbard, Arctic.

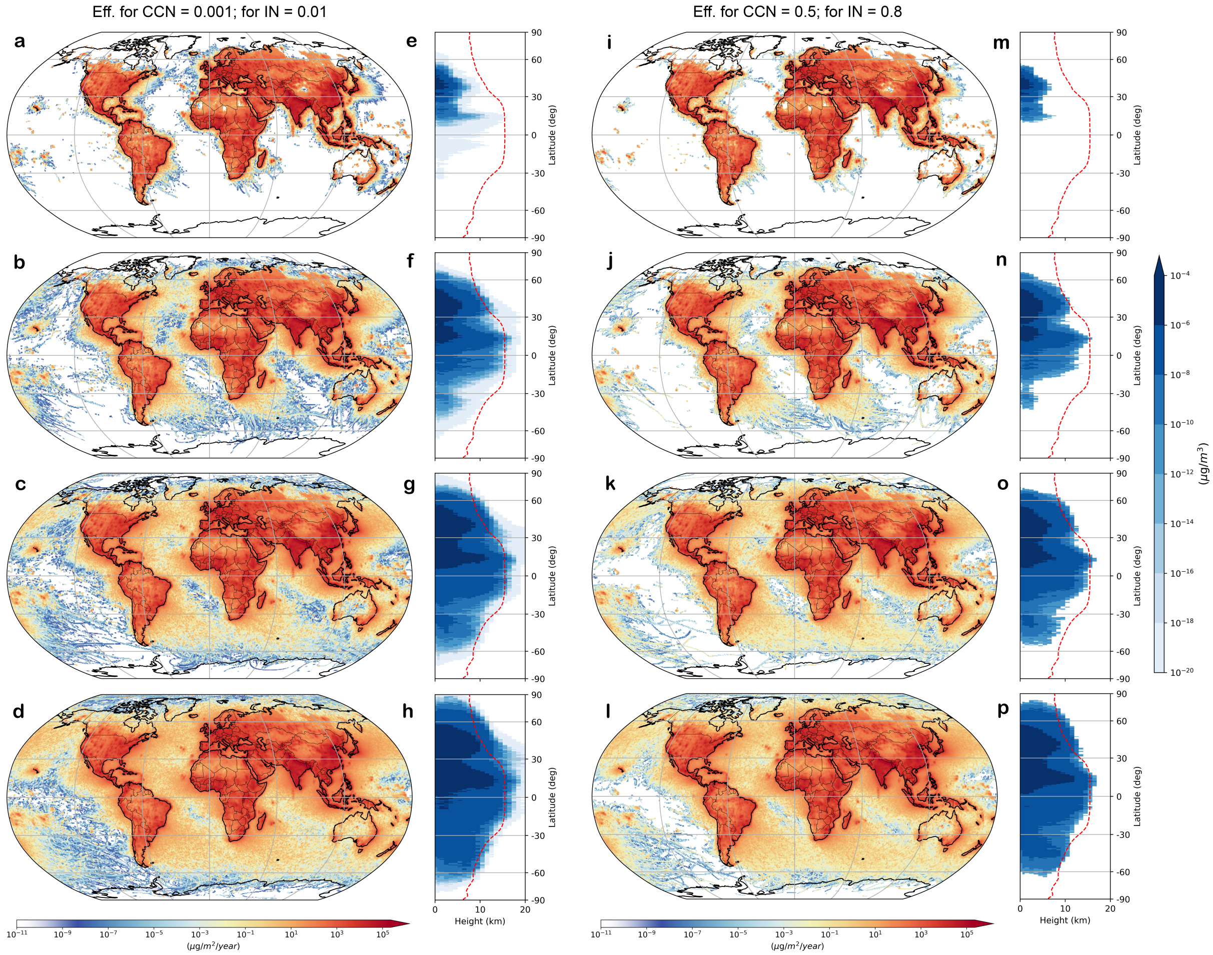

Figure S 10: **Shape dependence of hydrophobic (left two columns) and hydrophilic (right two columns) microplastic particle deposition and vertical transport.** Shown is the simulated total deposition in 2018 for spheres (**a,i**) and straight cylindric fibers with identical volume and AR=20 (**b,j**), AR=50 (**c,k**), and AR=100 (**d,l**) and the zonal median value of mass concentration for spheres (**e,m**) and straight cylindric fibers with identical volume and AR=20 (**f,n**), AR=50 (**g,o**), and AR=100 (**h,p**). Emissions are based on the population density map. Dashed red line indicates the tropopause height.

Table S 1: Chain of equations to calculate the drag coefficient C_d for three types of orientation based on the simplified shape correction scheme of Bagheri and Bonadonna (2016).[1] Re is the Reynolds number, k_S is Stokes' and k_N is Newton's drag corrections, F_S is Stokes' form factor, F_N is Newton's form factor, d_{eq} is the diameter of a sphere of equivalent volume, and α, β are empirical expressions.

Random orientation	Horizontal orientation	Average orientation
$F_S = fe^{1.3}$	$F_S = fe^{1.3}$	$F_S = fe^{1.3}$
$F_N = f^2 e$	$F_N = f^2 e$	$F_N = f^2 e$
$\rho' = \frac{\rho_p}{\rho_f}$	$\rho' = \frac{\rho_p}{\rho_f}$	$\rho' = \frac{\rho_p}{\rho_f}$
$\alpha = 0.45 + 10/(\exp(2.5 \log \rho') + 30)$		
$\beta = 1 - 37/(\exp(3 \log \rho') + 100)$		
$k_{S,rand} = (F_S^{1/3} + F_S^{-1/3})/2$	$k_{S,hor} = 0.5(F_S^{0.05} + F_S^{-0.36})$	$k_{S,average} = (k_{S,rand} + k_{S,hor})/2$
$k_{N,rand} = 10^{\alpha[-\log(F_N)]^{\beta}}$	$k_{N,hor} = 10^{0.77[-\log(F_N)]^{0.63}}$	$k_{N,average} = (k_{N,rand} + k_{N,hor})/2$

$$C_d = \frac{24k_S}{Re}(1 + 0.125(Re \cdot k_N/k_S)^{2/3}) + \frac{0.46k_N}{1+5330/(Re \cdot k_N/k_S)}$$

Table S 2: Percentage difference between model values for three types of particle orientation and experimental results for the three shapes studied and averaged over all sizes per shape.Values are expressed as mean relative difference ± standard deviation.

	Random orientation	Horizontal orientation	Average orientation
Straight fiber	16.8±8.4%	13.3±10.0%	8.6±8.2%
Semicircular fiber	13.4±4.7%	12.0±6.6%	6.8±3.3%
Quarter circular fiber	17.1±4.4%	7.9±5.6%	4.4±4.0%

Table S 3: Deposited mass of microplastic fibers in different regions of the globe (in tonnes per year).

		High Arctic (>75 °)	Southeast Pacific Ocean	Central Indian Ocean	Central Australia	World Ocean
Hydrophobic microplastics	Sphere	0.00	0.73	11.23	1.02	16201.51
	Straight fiber AR = 20	0.16	1.53	29.61	1.16	18359.41
	Straight fiber AR = 50	0.46	2.46	52.20	1.28	20312.82
	Straight fiber AR = 100	2.38	4.29	78.61	1.94	22547.51
Hydrophilic microplastics	Sphere	0.00	1.33	40.17	1.03	16236.26
	Straight fiber AR = 20	0.03	1.15	53.79	1.14	18071.91
	Straight fiber AR = 50	0.35	1.34	69.16	1.29	19856.23
	Straight fiber AR = 100	1.58	2.68	86.38	1.57	21889.10

References

(1) Bagheri, G.; Bonadonna, C. On the drag of freely falling non-spherical particles. *Powder Technology* **2016**, 526–544.
